# *Trypanosoma* Species in Small Nonflying Mammals in an Area With a Single Previous Chagas Disease Case

**DOI:** 10.3389/fcimb.2022.812708

**Published:** 2022-02-11

**Authors:** Maria Augusta Dario, Cristiane Varella Lisboa, Samanta Cristina das Chagas Xavier, Paulo Sérgio D’Andrea, André Luiz Rodrigues Roque, Ana Maria Jansen

**Affiliations:** ^1^ Laboratory of Trypanosomatid Biology, Oswaldo Cruz Institute, Oswaldo Cruz Foundation, Rio de Janeiro, Brazil; ^2^ Laboratory of Biology and Parasitology of Wild Reservoir Mammals, Oswaldo Cruz Institute, Oswaldo Cruz Foundation, Rio de Janeiro, Brazil

**Keywords:** Trypanosomatidae, mammalian host, *Trypanosoma cruzi* clade, infection, Atlantic Forest

## Abstract

Trypanosomatids are hemoflagellate parasites that even though they have been increasingly studied, many aspects of their biology and taxonomy remain unknown. The aim of this study was to investigate the *Trypanosoma* sp. transmission cycle in nonflying small mammals in an area where a case of acute Chagas disease occurred in Mangaratiba municipality, Rio de Janeiro state. Three expeditions were conducted in the area: the first in 2012, soon after the human case, and two others in 2015. Sylvatic mammals were captured and submitted to blood collection for trypanosomatid parasitological and serological exams. Dogs from the surrounding areas where the sylvatic mammals were captured were also tested for *T. cruzi* infection. DNA samples were extracted from blood clots and positive hemocultures, submitted to polymerase chain reaction targeting SSU rDNA and gGAPDH genes, sequenced and phylogenetic analysed. Twenty-one wild mammals were captured in 2012, mainly rodents, and 17 mammals, mainly marsupials, were captured in the two expeditions conducted in 2015. Only four rodents demonstrated borderline serological *T. cruzi* test (IFAT), two in 2012 and two in 2015. *Trypanosoma janseni* was the main *Trypanosoma* species identified, and isolates were obtained solely from *Didelphis aurita*. In addition to biological differences, molecular differences are suggestive of genetic diversity in this flagellate species. *Trypanosoma* sp. DID was identified in blood clots from *D. aurita* in single and mixed infections with *T. janseni*. Concerning dogs, 12 presented mostly borderline serological titers for *T. cruzi* and no positive hemoculture. In blood clots from 11 dogs, *T. cruzi* DNA was detected and characterized as TcI (n = 9) or TcII (n = 2). Infections by *Trypanosoma rangeli* lineage E (n = 2) and, for the first time, *Trypanosoma caninum*, *Trypanosoma dionisii*, and *Crithidia mellificae* (n = 1 each) were also detected in dogs. We concluded that despite the low mammalian species richness and degraded environment, a high *Trypanosoma* species richness species was being transmitted with the predominance of *T. janseni* and not *T. cruzi*, as would be expected in a locality of an acute case of Chagas disease.

## Introduction

Trypanosomatids are obligate parasites capable of infecting invertebrates, vertebrates, and plant hosts (Hoare, 1966; Vickerman, 1976). To date, 25 genera are described within this order and are classified according to the number of hosts involved in the development of their life cycle (d'Avila-Levy et al., 2015; Maslov et al., 2019; Kostygov et al., 2020; Lukeš et al., 2021): a) a group called monoxenic parasites formed by species that classically have only one definitive host, the invertebrate animals; and b) a second group called heteroxenic that have two hosts to complete their life cycle, an invertebrate animal and the other can be a vertebrate animal or a plant. Nineteen genera are recognized as tripanosomatid monoxenous (d'Avila-Levy et al., 2015; Kaufer et al., 2017; Kostygov et al., 2020; Lukeš et al., 2021): *Angomonas*, *Blastocrithidia*, *Blechomonas*, *Crithidia*, *Herpetomonas*, *Kentomonas*, *Jaenimonas*, *Lafontella*, *Leptomonas*, *Lotmaria*, *Novymonas*, *Obscuromonas*, *Paratrypanosoma*, *Rhychoidomonas*, *Sergeia*, *Strigonomonas*, *Vickermania*, *Wallacemonas*, and *Zelonia*. Its definitive hosts are insects of the orders Diptera, Hemiptera, and Siphonaptera, such as mosquitoes, flies, bees, and fleas (Kozminsky et al., 2015). Six genera are classified as heteroxenous trypanosomatids (d'Avila-Levy et al., 2015; Kaufer et al., 2017; Maslov et al., 2019): *Endotrypanum*, *Leishmania*, *Paraleishmania*, *Porcisia*, and *Trypanosoma*, which infect vertebrate animals, and *Phytomonas*, which is capable of infecting plants. The genera *Leishamania* and *Trypanosoma* infect a diversity of species of vertebrate animals and are the most studied genera due to their medical and economic importance (Simpson et al., 2006).

The clade denominated *Trypanosoma cruzi* is composed by, at least, nine different species and other operational taxonomic units that infect a broad range of mammalian species (Lopes et al., 2018; Clément et al., 2019; Rodrigues et al., 2019; Alves et al., 2021) and, from whose the transmission cycle is known, are transmitted by hematophagous insects, such as cimicids and triatomines (Gardner and Molyneux, 1988a; Jansen et al., 2018). The species included in this clade are distributed on five continents (Asia, Africa, Oceania, Europe, and the Americas) (Noyes et al., 1999; Hamilton et al., 2009; Hamilton et al., 2012; Lima et al., 2012; Lima et al., 2013; Lima et al., 2015; Barbosa et al., 2016; Botero et al., 2016; Lopes et al., 2018; Mafie et al., 2018; Wang et al., 2019). According to phylogenetic analysis, the following species are classified into this group: i) *T. livingstonei*, a species described in African bats (Lima et al., 2013); ii) *T. noyesi* species described in Australian marsupials (Botero et al., 2016); iii) *T. janseni*, species first described in marsupials of the species *Didelphis aurita* in the Atlantic Forest of Brazil (Lopes et al., 2018); iv) *T. wauwau* and *Trypanosoma* sp. neobats described in neotropical bat species from Latin America (Lima et al., 2015); v) *T. vespertilionis* described in European and African bats (Hamilton et al., 2012); vi) *T. conorhini* a cosmopolitan species described in rodents (Hamilton et al., 2012); vii) three isolated representatives of a monkey, a civet and an African bat (Hamilton et al., 2009); viii) *T. teixeirae* described in an Australian bat (Barbosa et al., 2016); ix) *T. rangeli*, a mammalian multihost species in Latin America (Stevens et al., 1999); and x) species of the subgenus *Schizotrypanum*: *T. cruzi*, *T. dionisii*, and *T. erneyi* (Lima et al., 2012). *Trypanosoma cruzi*, the etiological agent of Chagas disease (CD) in humans, is a multihost parasite that infects at least eight mammalian orders, and it is transmitted by triatomines (Jansen et al., 2018). It presents a genetic diversity in which seven genotypes, denominated as discrete typing units (DTUs), TcI to TcVI and Tcbat, are recognized (Zingales et al., 2009; Zingales et al., 2012). Indeed, an increasing number of new species and new host–parasite interactions have been recently discovered within the family, especially when studying wild-free-ranging mammals. Even *T. cruzi*, which is a well-studied parasite, still demonstrates gaps in the knowledge of its ecology, transmission, and epidemiology. Exactly this point gave rise to this study.

The aim of this study was to investigate *Trypanosoma* sp. infection in small nonflying mammals in the Atlantic Forest of Mangaratiba municipality, Rio de Janeiro state, southeast Brazil. For this purpose, three excursions were carried out: one in 2012 and two in 2015. Thus, our objective was to monitor possible changes in the enzootic scenario of *Trypanosoma* spp. infection in nonflying small mammals in the area. Our intent was to assess whether there was an alteration in the profile of the enzooty with the increase in the mammalian infection rate by *T. cruzi* or whether the enzooty remained stable.

## Material and Methods

### Ethical Statement

The capture of small sylvatic mammals was authorized by the Sistema de Autorização e Informação em Biodiversidade—SISBIO of the Instituto Brasileiro do Meio Ambiente e dos Recursos Naturais Renováveis (IBAMA)-(permanent license number 3365-1) and by the Instituto Estadual do Meio Ambiente (INEA/RJ) under license number 028/2015. Mammalian blood sample collection and euthanasia were performed according to the Ethical Committee for Animal Use of the Oswaldo Cruz Foundation (license LW-81-12).

### Study Area

**Figure 1:**
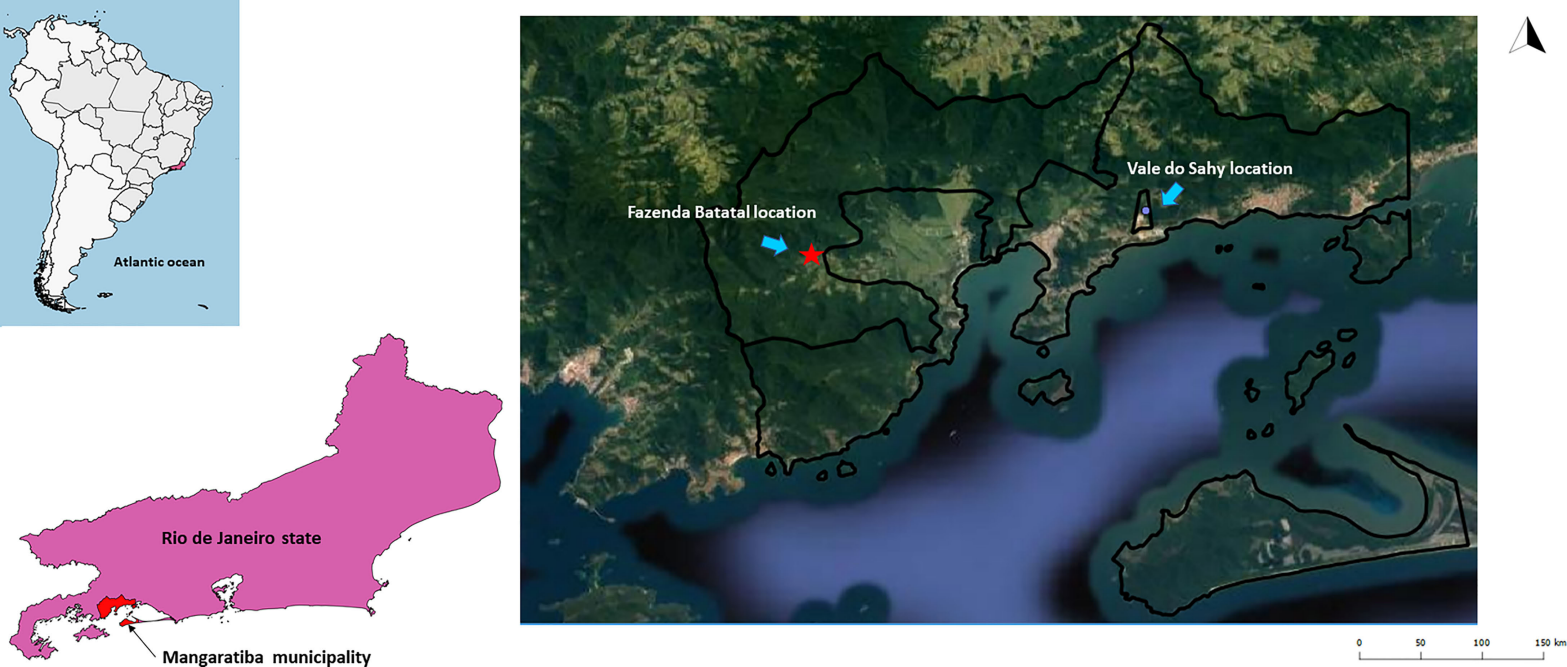
Mangaratiba municipality study locations in 2012 and 2015. The red star represents the area where acute CD occurred in 2012. The blue dot is where the Cunhambebe Park is located. Data source: Instituto Brasileiro de Geografia e Estatística—IBGE (www.ibge.gov.br); Google Earth (https://www.google.com.br/intl/pt-BR/earth/).

Mangaratiba municipality is located in the Rio de Janeiro Green Coast, 85 km from Rio de Janeiro municipality. It occupies a 356 km² area, and its estimated population was 41,557 inhabitants in 2016, according to the Instituto Brasileiro de Geografia e Estatística (IBGE). In 2012, an acute case of CD confirmed by parasitological, serological, and molecular tests was reported (Sangenis et al., 2015). The case was acquired through the contaminative route by a man who slept in a hammock. Two areas were chosen to investigate *Trypanosoma* spp. transmission cycle: i) Fazenda Batatal, where in 2012 the case of CD occurred, and ii) Vale do Sahy, where Cunhambebe Park is located approximately 15 km from where the acute CD occurred (**Figure 1**).

### Small Sylvatic Mammals’ Capture

On the first expedition in 2012 (dry season—August), three linear transects were established in the Fazenda Batatal location: i) two close to the place where the man became infected and ii) one in a forest fragment farther away but still within the same region. Sherman^®^ (H. B. Sherman Traps, Tallahassee, FL, USA) and Tomahawk^®^ (Tomahawk Live Traps, Tomahawk, WI, USA) type traps were placed on the ground and/or understory and baited with universal bait (Dario et al., 2016). Two surveys were performed in 2015 at Fazenda Batatal and Vale do Sahy locations in the wet (March) and dry (September) seasons employing the same capture methodology. The capture effort was 440 traps for 4 nights in 2012 and 480 traps for 4 nights in each of the 2015 expeditions.

For all the animals, morphological characteristics and body measurements were recorded for taxonomic identification. The identification of rodents was confirmed by karyological analyses (Bonvicino et al., 2005). Only rodent specimens that required taxonomical confirmation obtained by karyotyping were sacrificed and the carcasses were deposited as voucher specimens in the collection of the Nacional Museum (Universidade Federal do Rio de Janeiro, UFRJ, Rio de Janeiro, Brazil). All captured animals were manipulated according to the safety manual for the use of wild mammals in research (Dario et al., 2016) and were anesthetized (9:1 ketamine chlorhydrate 10% and acepromazine 2%) for blood sample collection (by cardiac puncture) for parasitological and serological exams.

### Dog Survey

An active search for dogs was conducted in the residences near the locations where the sylvatic mammals were captured in both years. With the informed consent of their owners, blood samples were collected using Vacutainer^®^ tubes containing EDTA by puncturing the femoral vein of the dog. A questionnaire was used to record the name, age, sex, size, and primary function (hunting, companionship, or protection) and anatomical peculiarities. Dogs from the same house were considered to be a single event.

### Trypanosomatid Infection Survey

Parasitological and serological methods were used to identify *Trypanosoma* species infection in sylvatic mammals and dogs. The parasitological methods included (i) fresh blood examination and (ii) hemocultures (300 µl each of animal blood in two tubes containing Novy McNeal-Nicole (NNN) supplemented with Liver Infusion Tryptose (LIT) overlay supplemented with 10% fetal bovine serum and 140 mg/ml antibiotic). Hemocultures were examined fortnightly for five months. Positive cultures, which demonstrated parasite growth, were amplified, cryopreserved, and deposited in the Coleção de *Trypanosoma* de Mamíferos Silvestres, Domésticos e Vetores, COLTRYP/Fiocruz. The liquid phase of initially positive hemocultures, in which the flagellate did not thrive successfully, was centrifuged at 4,000*g*, and the resultant sediments were stored at −20°C for molecular identification. In addition, mammalian whole blood was centrifuged at 1,180*g* for 15 min, and the blood clot was used in the molecular analysis (Alves et al., 2021).

Serological analyses were performed by indirect immunofluorescence antibody test (IFAT) to detect IgG antibodies in sera of sylvatic mammals and dogs (Camargo, 1966). Reference strains I00/BR/00F (TcI) and MHOM/BR/1957/Y (TcII) from axenic cultures were mixed in equal (1:1) proportions and used as antigens. The sera from Murinae rodents and plasma from dogs were tested with anti-rat IgG and anti-dog IgG, respectively, conjugated with fluorescein isothiocyanate (Sigma, St. Louis, MO, USA). The marsupial sera were tested as described in (Xavier et al., 2014) using an in-house anti-*Didelphis* spp. IgG and the reactions revealed using an anti-rabbit IgG also conjugated with fluorescein isothiocyanate. The cutoff values for the IFAT were 1:40 for marsupials and dogs and 1:10 for rodents (Herrera et al., 2005). To confirm the serological results of the dogs, an enzyme-linked immunosorbent assay (ELISA-Bio-Manguinhos, FIOCRUZ, Rio de Janeiro, RJ, Brazil) was performed. The cutoff value for ELISA was an optical absorbance ≥0.200, mean ±3 standard deviations. In addition, two negative and two positive control sera were added to each reaction of the dog. For the IFAT assays in wild mammals, specific positive and negative controls were also added according to each mammalian family.

To exclude cross-reactions between *T. cruzi* and *Leishmania* sp., an IFAT using a mixture of axenic cultures of *L. infantum* (IOC/L579—MHOM/BR/1974/PP75) and *L*. *braziliensis* (IOC/L566—MHOM/BR/1975/M2903), an ELISA and a rapid test for the diagnosis of canine visceral leishmaniasis (CVL) (TR DPP^®^, Bio-Manguinhos, FIOCRUZ, Rio de Janeiro, RJ, Brazil) were performed. Serological positivity criteria for dog samples were obtained according to Xavier et al. (2012): dogs with serological titers higher for *Leishmania* sp. than for *T. cruzi* were considered infected only for *Leishmania* sp. when *T. cruzi* titers were ≤1∶80 and as mixed infected when titers were >1∶80.

### Trypanosomatid Species Molecular Characterization

Total genomic DNA from positive hemocultures and blood clots was extracted using phenol–chloroform (Sambrook and Russel, 2001) and ammonium acetate precipitation (Rodrigues et al., 2019). For *Trypanosoma* species identification, nested polymerase chain reaction (nested-PCR) for the small subunit ribosomal RNA gene (SSU rDNA) (external primers TRY927F 5’CAGAAACGAAACACGGGAG3’ and TRY927R 5’CCTACTGGGCAGCTTGGA3’; internal primers SSU561F 5’TGGGATAACAAAGGAGCA3’ and SSU561R 5’CTGAGACTGTAACCTCAAAGC3’) (Noyes et al., 1999; Smith et al., 2008) was performed for positive hemocultures and blood clot DNA, while a convencional PCR for glycosomal glyceraldehyde 3-phosphate dehydrogenase (gGAPDH) gene (GAPTRY-mod F 5'GGBCGCATGGTSTTCCAG3' and GAPTRYr R 5'CCCCACTCGTTRTCRTACC3') (Borghesan et al., 2013) was performed only for hemoculture samples. *Trypanosoma. cruzi* strain SylvioX/10cl1 was used as a positive control, and we included one reaction with distilled water instead of DNA as a negative control.

The PCR products (~650 bp for the SSU rDNA gene and ~800 bp for the gGAPDH gene) were visualized using a 2% agarose gel stained with ethidium bromide and purified according to the manufacturer’s instructions (Illustra GFX PCR DNA and gel band purification kit—GE Healthcare Life Sciences, Little Chalfont, Buckinghamshire, UK). Both strands of DNA were then sequenced using a BigDye Terminator v3.1 Cycle Sequencing kit (Applied Biosystems, Foster City, CA, USA) on an ABI 3730 DNA sequencer available at the PDTIS/Fiocruz sequencing platform. To obtain SSU rDNA and gGAPDH consensus sequences, DNA strands were assembled using SeqMan (DNASTAR Lasergene, Gatc, Konstanz, Germany) and then edited using MegaX (Kumar et al., 2018). The SSU rDNA and gGAPDH sequences from hemoculture isolates were concatenated for phylogenetic analysis to increase the robustness of the results.

Trypanosomatid sequences from this study and others retrieved from GenBank were aligned using the algorithmL-INS-i in MAFFT v.7.0 (Katoh and Standley, 2013). Phylogenetic reconstructions by Bayesian inference (BI) and maximum-likelihood (ML) methods were performed using the Mrbayes (Huelsenbeck and Ronquist, 2001; Ronquist and Huelsenbeck, 2003) and IQ-tree (Nguyen et al., 2015) programs, respectively. The best nucleotide substitution models were chosen according to the corrected Akaike information criterion (cAIC) in ModelFinder (Kalyaanamoorthy et al., 2017) for each phylogenetic analysis. All the programs were available on the Phylosuite v1.2.2 platform (Zhang et al., 2020). For the ML inference branch support, ultrafast bootstrapping (Hoang et al., 2018) with 5,000 replicates with 1,000 maximum interactions and 0.99 minimum correlation coefficients and the SH-aLRT branch test with 5,000 replicates were applied. In the BI, the Bayesian Markov chain Monte Carlo (MCMC) method was used to assign trypansomatids prior to information. Four independent runs were performed for 20 M with sampling every 2,000 generations with 25% burn-in from each run. The final trees were visualized on Figtree v 1.4.3.

## Results

The enzootic scenario of *Trypanosoma* spp. transmission in the area remained stable and different from all other areas that reported cases and outbreaks of acute CD. We observed a low richness of sylvatic mammal species and low *T. cruzi* transmission in this region of the Atlantic Forest. However, we observed an important diversity of trypanosomatid species being transmitted in the area, mainly among dogs.

### Small Sylvatic and Synanthropic Mammals’ Occurrence

In the first expedition in 2012, 21 mammal specimens were captured, mainly rodents, in Fazenda Batatal, which is where the case happened: *Akodon cursor* (n = 13), *Oxymycterus judex* (n = 3), *Oligoryzomys nigripes* (n = 2), *Euryzygomatomyz americanatus* (n = 1), *Nectomys squamipes* (n = 1), and *Didelphis aurita* (n = 1). The capture success was of 4.8%. During the two expeditions performed in 2015, 15 small sylvatic mammals (seven individuals in March and eight individuals in September) were captured and identified into four species: *D. aurita* (n = 10), *Marmosa paraguayana* (n = 1), *A. cursor* (n = 3), and *Euryoryzomys russatus* (n = 1). Two synanthropic rodent species, *Rattus rattus* and *Rattus norvegicus*, were also captured (**Table 1**). The capture success was 3.54%. Comparing the three expeditions, we observed a change in mammalian species captured. In 2012, the predominance of rodent species (n = 20; 90.9%) was observed, and in 2015, *D. aurita* predominance (n = 10; 58.8%) was observed in both locations.

**Table 1:** Small sylvatic and synanthropic mammals captured and *Trypanosoma* sp. infection in Mangaratiba municipality, RJ state in 2015.

Location	LBCE	COLTRYP	Species	Molecular characterization		GenBank accession number		
				Hemoculture	Blood clot	SSU rDNA hemoculture	gGAPDH hemoculture	SSU rDNA blood clot
Fazenda Batatal	17242	–	*Marmosa paraguayana*	*–*	–	–	–	Not performed
	17245	–	*Akodon cursor*	*–*	–	–	–	Not performed
	17246	00608	*Didelphis aurita*	*T. janseni*	*Trypanosoma* sp. DID	OL314519	OL314513	OL314521
	17247	00607	*Didelphis aurita*	*T. janseni*	*T. janseni*	OL314518	OL314512	OL314525
	17248	00604	*Didelphis aurita*	*T. janseni*	–	OL314515	OL314509	Not performed
	17250	–	*Akodon cf. cursor*	*–*	*T. janseni*	–	–	OL314524
	17251	–	*Rattus norvegicus*	*–*	–	–	–	Not amplified
	17254	–	*Akodon cf. cursor*	*–*	–	–	–	Not amplified
	18335	–	*Didelphis aurita*	*–*	*Trypanosoma* sp. DID	–	–	MH404845*
Vale do Sahy	17249	–	*Rattus rattus*	*–*	–	–	–	Not performed
	17243	00605	*Didelphis aurita*	*T. janseni*	–	OL314516	OL314510	Not performed
	17244	00606	*Didelphis aurita*	*T. janseni*	–	OL314517	OL314511	Not performed
	17252	–	*Didelphis aurita*	*–*	–	–	–	Not amplified
	17253	–	*Didelphis aurita*	*T. janseni*	*Trypanosoma* sp. DID	OL314520	OL314514	OL314523
	18336	–	*Didelphis aurita*	*–*	–	–	–	Not amplified
	18337	–	*Didelphis aurita*	*–*	*Trypanosoma* sp. DID	–	–	OL314522
	18338	–	*Euryoryzomys russatus*	*–*	*T. janseni*	–	–	OL314526

*Sequence deposited and published by Rodrigues et al. (2019).

### Parasitological and Serological Survey in Small Sylvatic and Synanthropic Mammals and Domestic Dogs for *Trypanosoma* spp. Infection

**Figure 2:**
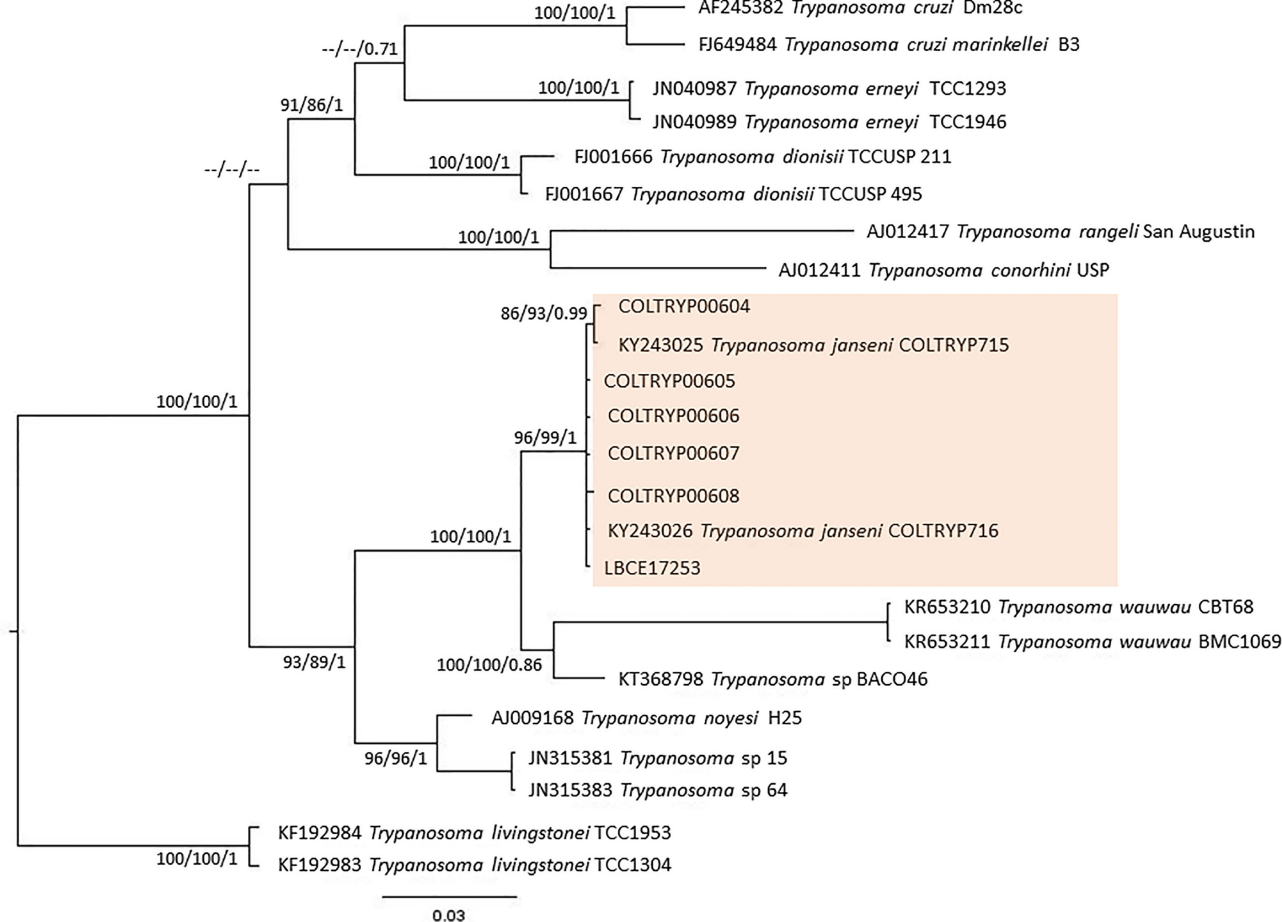
Concatenated (SSU rDNA + gGAPDH) *T. cruzi* clade phylogenetic tree based on 1121 base pair fragment lengths of sylvatic mammal hemocultures. The tree was inferred using transitional3 equal frequencies plus gamma distribution (TIM3e+G) and generalized time reversible with invariant sites and amino acid frequencies plus gamma distribution (GTR+F+I+G) models for ML and BI, respectively. The numbers at nodes correspond to ML (ultrabootstrap, SH-aLRT) and BI (posterior probability). The scale bar shows the number of nucleotide substitutions per site. The red square represents group formed *T. janseni* sequences from different culture isolation.

None of the parasitological exams (fresh blood and hemoculture) performed in the sylvatic mammals captured in 2012 were positive. In contrast, of the 17 hemocultures performed in 2015, six from *D. aurita* were positive (**Table 1**): five of them were successfully amplified, one failed to obtain parasite amplification, and molecular characterization was performed in the culture sediment (LBCE17253). The DNA sequences obtained from SSU rDNA and gGAPDH were identified as *T. janseni.* In the SSU rDNA sequence alignment, a single nucleotide polymorphism (SNP) was observed at site 975 between COLTRYP00604, KY243025 and the other *T. janseni* sequences. According to the phylogenetic analysis (**Figure 2**), the samples formed two groups of *T. janseni*. Serologically, all marsupials were negative, and four rodents were positive for *T. cruzi* infection: two *O. judex* (LBCE18303 and LBCE18316—serological titer 1:20) in 2012 and two synanthropic rodents (*R. novergicus* LBC17251 and *R. rattus* LBCE17249—serological titer 1:40) in 2015.

The forms with dog data filled before blood collection showed that we were not dealing with the same animal already examined in a previous excursion. Actually, the turnover rate of dogs in the rural areas of the country is significant. In addition, only adult dogs were examined. All fresh blood examinations and hemocultures were negative for *Trypanosoma* infection in the dogs from the Fazenda Batatal (n = 21—2012; n = 26—2015) and Vale do Sahy (n = 32) areas that were examined. Twelve dogs were seropositive for *T. cruzi* infection (**Table 2**).

**Table 2:** Trypanosomatidae infection in dogs (serological tests for *T. cruzi* and molecular blood clot for trypanosomatids) in Fazenda Batatal and Vale do Shahy locations, Mangaratiba municipality, Rio de Janeiro state.

Year	LBT	Location	IFAT	ELISA	Blood clot	GenBank accession number
2012	3476	Fazenda Batatal	1:80	Positive	Not performed	–
2015	6212	Fazenda Batatal	1:80	Positive	Not amplified	–
2015	6213	Fazenda Batatal	1:40	Bordeline	*T. cruzi* TcI	OL314533
2015	6215	Fazenda Batatal	1:80	Positive	Not amplified	–
2015	6218	Fazenda Batatal	1/20	Positive	*T. cruzi* TcI	OL314534
2015	6219	Fazenda Batatal	1:160	Positive	Not amplified	–
2015	6701	Fazenda Batatal	1:20	Negative	*T. cruzi* TcI	OL314530
2015	6702	Fazenda Batatal	1:80	Positive	*T. caninum*	OL314539
2015	6704	Fazenda Batatal	1:40	Negative	*C. mellificae*	OL314540
2015	6705	Fazenda Batatal	1:20	Negative	*T. rangeli*	MN661344*
2015	6706	Fazenda Batatal	1:20	Negative	*T. rangeli*	MN661345*
2015	6707	Fazenda Batatal	1:20	Negative	*T. cruzi* TcI	OL314529
2015	6226	Vale do Sahy	1:20	Positive	*T. cruzi* TcI	OL314537
2015	6228	Vale do Sahy	1:40	Negative	*T. dionisii*	OL314538
2015	6229	Vale do Sahy	Negative	Negative	*T. cruzi* TcI	OL314532
2015	6230	Vale do Sahy	1:80	Positive	*T. cruzi* TcI	OL314531
2015	6234	Vale do Sahy	1:20	Positive	*T. cruzi* TcII	OL314536
2015	6240	Vale do Sahy	1:80	Positive	Not amplified	–
2015	6241	Vale do Sahy	1:160	Positive	Not amplified	–
2015	6244	Vale do Sahy	1:160	Positive	*T. cruzi* TcII	OL314535
2015	6245	Vale do Sahy	1:80	Positive	Not amplified	–
2015	6709	Vale do Sahy	1:80	Negative	*T. cruzi* TcI	OL314528
2015	6711	Vale do Sahy	Not performed	Not performed	*T. cruzi* TcI	OL314527
2015	6715	Vale do Sahy	1:80	Positive	Not amplified	–
2015	6716	Vale do Sahy	1:80	Positive	Not amplified	–

*Sequence previously published by Dario et al. (2021a).

**Figure 3:**
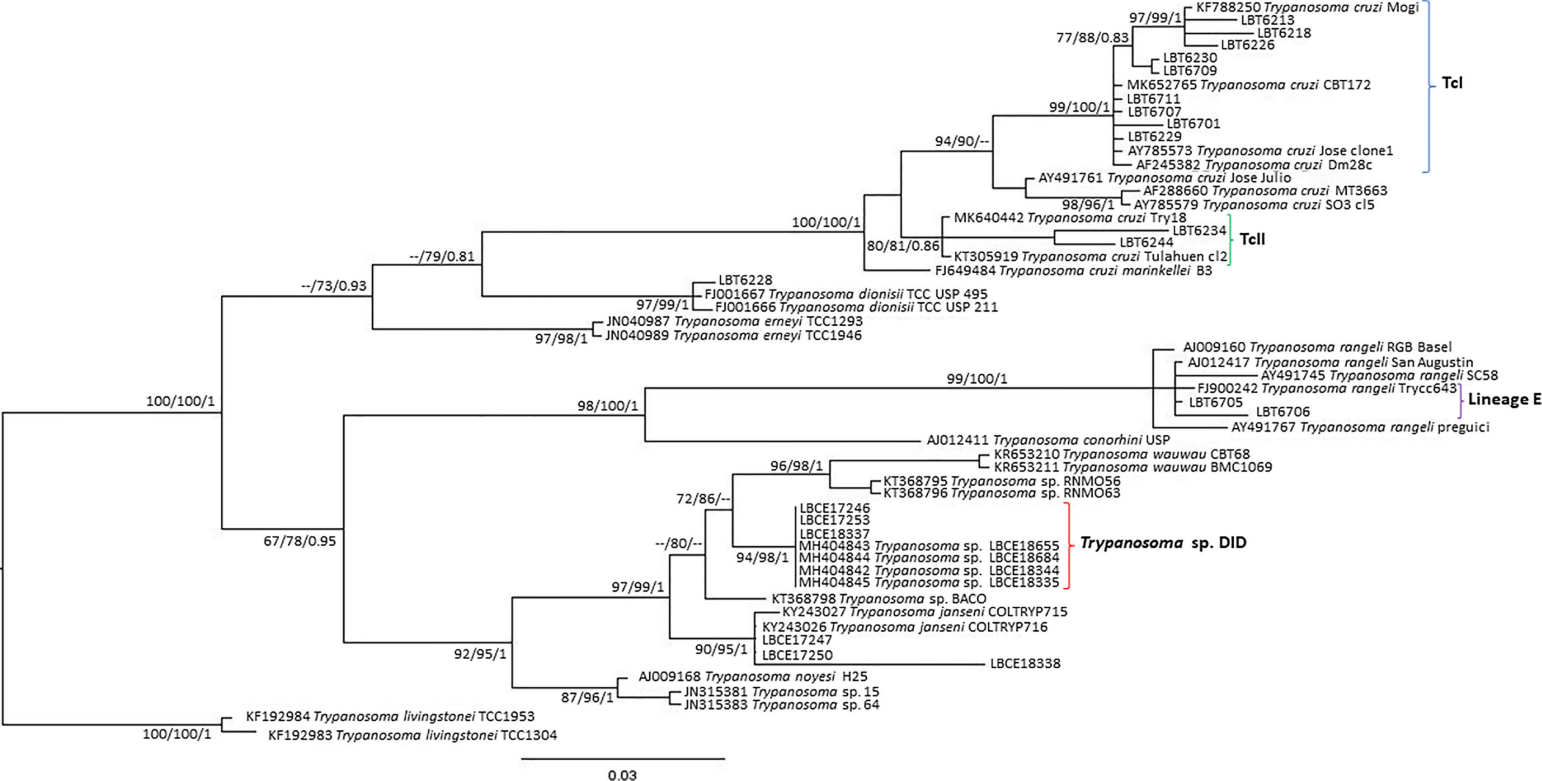
*Trypanosoma cruzi* clade phylogenetic tree based on 692 base pair SSU rDNA fragment lengths from sylvatic mammal and dog blood clots. The tree was inferred using transitional3 with amino acid frequencies and freeRate heterogeneity (TIM3+F+R2) and generalized time reversible with amino acid frequencies plus gamma distribution (GTR+F+G) models for ML and BI, respectively. The numbers at nodes correspond to ML (ultrabootstrap and SH-aLRT) and BI (posterior probability). The scale bar shows the number of nucleotide substitutions per site. The blood clot samples grouped with *T. cruzi, T. rangeli, Trypanosoma* sp. DID and *T. janseni*. The blue curly brackets represent the sequences that grouped with *T. cruzi* TcI sequences. The green curly brackets represent the sequences that grouped with *T. cruzi* TcII/TcVI sequences. The purple curly brackets represent the sequences that grouped with *T. rangeli* lineage E sequences. The red curly brackets represent the sequences that grouped with *Trypanosoma* sp. DID sequences.

**Figure 4:**
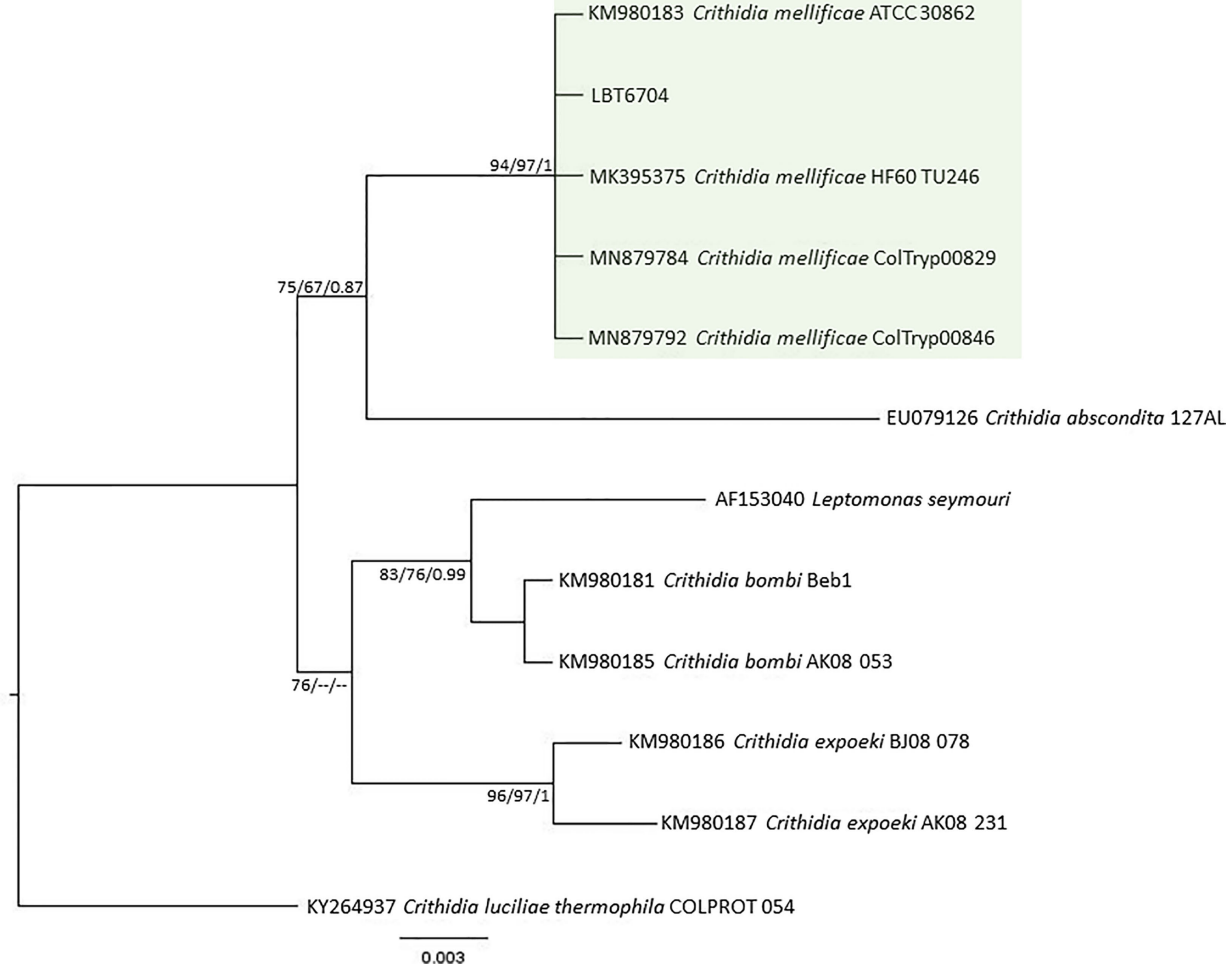
*Crithidia mellificae* phylogenetic tree based on 528 base pairs SSU rDNA fragment length from dog blood clot. The tree was inferred using transitional3 equal frequencies plus gamma distribution (TIM3e+G) and symmetrical plus gamma distribution (SYM+G) models for ML and BI, respectively. The numbers at nodes correspond to ML (ultrabootstrap and SH-aLRT) and BI (posterior probability). The scale bar shows the number of nucleotide substitutions per site. The green square represents the clade formed by LBT6704 with other *C. mellifiace* sequences from insects and mammals.

**Figure 5:**
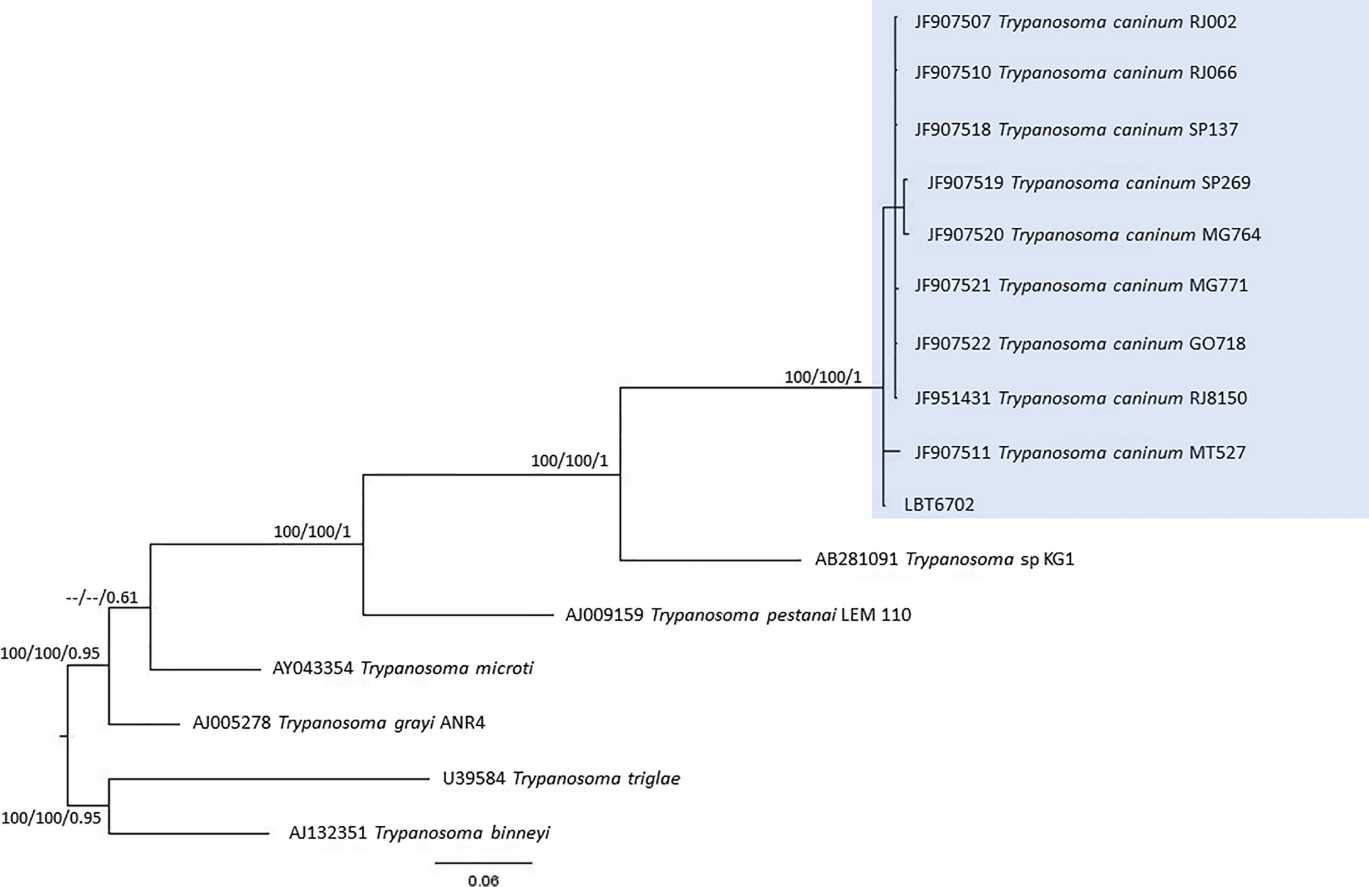
*Trypanosoma caninum* phylogenetic tree based on 605 base pair SSU rDNA fragment lengths from dog blood clots. The tree was inferred using transitional with amino acid frequencies and freeRate heterogeneity (TIM+F+R2) and symmetrical plus gamma distribution (SYM+G) models for ML and BI, respectively. The numbers at nodes correspond to ML (ultrabootstrap, SH-aLRT) and BI (posterior probability). The scale bar shows the number of nucleotide substitutions per site. The blue square represents the clade formed by LBT6702 with other *T. caninum* sequences from different Brazilian states.

Of the 17 specimens of small mammals captured in 2015, 11 blood clot samples were obtained for molecular characterization. Seven samples showed infection with trypanosomatids (**Table 1** and **Figure 3**), namely, *T. janseni* in *A. cursor* (n = 1), *E. russatus* (n = 1), *D. aurita* (n = 1), and *Trypanosoma* sp. DID in four *D. aurita* (**Table 1**). Two of the blood clot samples (LBCE17246 and LBCE17253) presented infection by *Trypanosoma* sp. DID, while in the hemoculture, molecular characterization showed that these samples were infected by *T. janseni* (**Figures 2**, **3** and **Table 1**). Regarding dogs, 16 blood clot samples demonstrated DNA of *C. mellificae* (n = 1) (**Figure 4**), *T. cruzi* DTU TcI (n = 9), *T. cruzi* DTU TcII (n = 2), *T. dionisii* (n = 1), and *T. rangeli* subpopulation E (n = 2) (**Figure 3**), and *T. caninum* (n = 1) (**Figure 5**). Comparing the results observed in the serological and blood clot molecular characterization, three samples characterized as *T. cruzi* belonged to dogs that presented serological titers and positive ELISA for *T. cruzi*: LBT6230, LBT6244, and LBT6702 (**Table 2**), but the sample LBT6702 presented infection by *T. caninum* in blood clot molecular characterization.

## Discussion

In this study, we demonstrated a peculiar enzootic transmission cycle of trypanosomatid species among sylvatic mammals and dogs from the Mangaratiba Atlantic Forest, where one case of acute CD had been diagnosed 3 years before. The enzootic scenario in relation to *T. cruzi* transmission did not change between 2012 and 2015. No sylvatic mammals presented infectivity competence for *T. cruzi* transmission. The difference observed was that more marsupials were captured in 2015 than in 2012, and some sylvatic mammals were infected by *T. janseni*. There are four aspects to be highlighted in this study that are uncommon in areas of CD: 1) there were very few reports of invasion of triatomines in houses; 2) there were no domiciled triatomines; 3) *T. cruzi* was the least prevalent trypanosomatid species in wild mammals; and 4) dogs were demonstrated to be infected by the higher diversity of Trypanosomatid species.

One of the species recently described within the *T. cruzi* clade is *T. janseni* (Lopes et al., 2018). This species was described from spleen and liver axenic cultures of a *D. aurita* marsupial of the Atlantic Forest of Rio de Janeiro state. Phylogenetically, this species was demonstrated to group together with *T. wauwau* and trypanosomatids from neotropical bats (Lopes et al., 2018). In this study, *T. janseni* was found to infect *D. aurita* and two rodent species: *A. cursor* and *E. russatus.* Three points suggest a possible biological diversity of *T. janseni*: i) by the ability to grow on NNN + LIT medium of some isolates in contrast to others probably representing a different genetic makeup (Berbigier et al., 2021); ii) sequencing showed a SNP, and the phylogenetic tree separated *T. janseni* into two groups. Obviously, the sequenced gene fragment is very small, and we would need whole 18S rDNA sequencing to evaluate this heterogeneity. Moreover, these data turn tempting to speculate that *T. janseni* also exhibits a certain degree of diversity, as is so common in these organisms. The high rates of marsupials infected by *T. janseni* observed in different Brazilian biomes reinforce that the genus *Didelphis* is one of the oldest hosts of parasite species from the *T. cruzi* clade and may also be the ancient host of *T. janseni* (Lopes et al., 2018; Rodrigues et al., 2019). However, *T. janseni* was demonstrated to be adaptable to other mammalian taxa, since it was identified in two rodent species (*A. cursor* and *E. russatus*) in this study and in Berbigier et al. (2021), and it was found infecting a dog, as demonstrated by PCR of blood clot samples (Malavazi et al., 2020).

Didelphids are known for their capacity to harbor several *Trypanosoma* species (Rodrigues et al., 2019). *Trypanosoma* sp. DID was described by Rodrigues et al. (2019) in *D. aurita* and *D. albiventris* from the Atlantic Forest and Cerrado biomes, respectively, and it is phylogenetically correlated to the clade formed by *T. wauwau*, *Trypanosoma* sp. neobats and *T. janseni*. Apparently, the set of results on *Trypanosoma* sp. DID shows one single taxonomic unit, since there is no genetic difference between the sequences, all grouped together. The occurrence of *Trypanosoma* sp. DID was already reported in mixed infections with *T. cruzi* (Nantes et al., 2021), and we observed this in *D. aurita* in single and mixed infections with *T. janseni*. Since they belong to the same clade, these findings raise the following questions: what is the possible mutual impact of a concomitant infection of *Trypanosoma* sp. DID with *T. janseni* in an opossum? Are these mixed infections with two or more species of *Trypanosoma* stable? These mixed infections demonstrate how important it is to use different diagnostic methodologies mainly if sylvatic mammals are studied and, once again, reinforce that mixed infections are the main infection pattern in nature.

The serological diagnosis for *T. cruzi* in *D. aurita* was reliable because no cross-reaction with *T. janseni* and *Trypanosoma* sp. DID was observed. However, there are some cases that deserve attention: i) the dog samples LBT6218 and LBT6701 were positive and negative in ELISA test, respectively, but displayed IFAT serological titers that were one dilution under the cutoff point (1:40), but their blood clot analyses demonstrated *T. cruzi* TcI. This could be explained by the paucity of parasites and maybe a more recent infection—we detected only DNA of *T. cruzi* and no positive parasitological testes ii) two dogs (LBT6213 borderline and LBT6704 negative ELISA tests), displayed cut off serological titers in IFAT (1:40). The identification of *C. mellificae* suggests cross reaction and shows the importance of always using two diagnostic tests in regard to diagnosing trypanosomatid infection.

Dogs exhibited the highest richness of trypanosomatid species infection, as demonstrated by blood clot molecular characterization: *T. cruzi* (DTUs TcI and TcII), *T. dionisii*, *T. caninum*, *T. rangeli*, and *C. mellificae*. *Crithidia mellificae*, a trypanosomatid classically referred to as a monoxenous insect parasite, is increasingly demonstrating itself as a generalist species capable of infecting an expressive number of mammal species (Rangel et al., 2019; Alves et al., 2021; Dario et al., 2021b). It is interesting to observe that *C. mellificae* is able to infect different species of mammals, and here, we include one more host, the domestic dog. The same for *T. dionisii*, classically described infecting bats (Gardner and Molyneux, 1988b), once again showed a broad mammalian host range since it has also been observed infecting dogs. This trypanosomatid species has also been described to infect marsupials, carnivores and humans (Dario et al., 2016; Rodrigues et al., 2019).

*Trypanosoma rangeli* is a multihost parasite transmitted by triatomines exclusively from the *Rhodnius* genus (Guhl and Vallejo, 2003) and is a heterogeneous parasite in which five lineages or two groups are recognized according to the molecular marker used: lineages A, B, C, D or E for nuclear markers (Maia da Silva et al., 2004; Maia da Silva et al., 2009) and KP1(+) or KP1(−) for kDNA (Vallejo et al., 2002). Concerning *T. rangeli*, we question ourselves how this parasite species is transmitted in the southeastern Atlantic Forest, since its transmission is related to *Rhodnius* triatomine species (Guhl and Vallejo, 2003), and we report its occurrence in an area where this genus is not reported, showing that there are many gaps in the knowledge of elementary aspects of the biology even of this species, one of the most studied within the family. Although Gurgel-Gonçalves et al. (2012) mentioned that *R. domesticus* occurs in the southeast Atlantic Forest, it is difficult to find the species. There is only one report about its occurrence in Espírito Santo state (Corrêa-do-Nascimento et al., 2020). Even when performing a search for triatomines in the region, the genera that probably we would have found were *Triatoma* and *Panstrongylus* and not the genus *Rhodnius*.

*Trypanosoma caninum*, a trypanosomatid species classified outside the *T. cruzi* clade, was described from intact skin samples from domestic dogs, and little is known about its biology. Numerous cases of natural dog infection by this *Trypanosoma* species have already been recorded in different regions of Brazil. Phylogenetic analyses revealed that all *T. caninum* isolates analyzed were grouped in the same cluster, regardless of geographic precedence or genetic marker used. Additionally, electronic and optical microscopy analysis showed the presence of atypical epimastigote forms, without free flagellum, in axenic cultures (Madeira et al., 2009; Barros et al., 2014; Barros et al., 2015). Here, we observed the encounter of *T. caninum* for the first time in a blood sample, which is not surprising since the genus *Trypanosoma* is defined as a hematozoan. The question is why, among so many dogs examined, this species has never been observed in peripheral blood and always in skin tissue (Madeira et al., 2009; Barros et al., 2012). *Trypanosoma caninum* is a trypanosomatid species described to date exclusively by isolation of intact fragments from domestic dogs in different Brazilian biomes (Madeira et al., 2009; Barros et al., 2012). Many aspects of this parasite species remain unknown, namely, their evolutionary forms in vertebrate hosts and possible vectors (Barros et al., 2012; Barros et al., 2014; Fonseca-Oliveira et al., 2018). Although *T. caninum* has never been isolated by hemoculture, we identified the infection in a dog blood clot sample. This was possible because blood clot molecular identification is a sensitive methodology, especially in identifying trypanosomatids that are difficult to isolate in culture media (Rodrigues et al., 2019).

Although showing the greatest richness of trypanosomes, dogs showed only cryptoparasitemias, since no blood culture was positive and the presence of trypanosomatid species in the blood could only be detected by PCR of blood clots. The set of results indicated indirect evidence of low infectivity in dogs for any trypanosomatid species in the three excursions. We have performed two different parasitological tests that demonstrated the infectivity potential of a mammal: i) fresh blood exam (direct test) and ii) hemoculture (indirect test), and both presented negative results for dogs. In fact, dogs rarely demonstrated high parasitemias by *T. cruzi* in Brazil (Jansen et al., 2017). These results showed that in the peripheral circulation, there is no circulating parasite and the chance of a triatomine specimen get infected in the blood meal is low, however, this would only be confirmed by performing vector feeding assays. We observed six dogs with positive IFAT for *T. cruzi* infection. One of these dogs even presented a titer of 1:160. These sera were also positive by ELISA, but these dogs were negative by blood clot PCR for *T. cruzi*. Positive blood clot PCRs for *T. cruzi* were observed in the dogs (n = 11) with lower serological tests, including four negative ELISA testing dogs, which probably reflects recent infections.

The aim of this study was to understand *T. cruzi* transmission in an area where there was a case of acute CD in Mangaratiba. However, to our surprise, infection by *T. cruzi* was uncommon among the captured mammals, as demonstrated by parasitological exams (molecular characterization) and by low serological titers in the three expeditions performed. We attempted to search for triatomines during the fieldwork and in the surrounding area where acute CD occurred; however, no specimens were found. Regarding triatomine occurrence in the area, the dwellers did not recognize triatomine insects or mention its occurrence. Only after our fieldwork, after we informed them about these insects, five triatomine specimens (one in 2012 and four in 2015) were reported and delivered by the residents (Roque ALR, personal communication; Dario MA, personal communication). All the specimens were identified as *Triatoma tibiamaculata*, and only one specimen (2012) was infected by *T. cruzi*, as detected by the intestinal content exam, and it was characterized as *T. cruzi* DTU TcI (Roque ALR, personal communication). We hypothesize that the infected *T. tibiamaculata* (Sangenis et al., 2015) involved in the human case probably became infected and moved further away from the study area. This observation raises the question concerning the displacement capacity of triatomines that, in addition to flying, can also travel by air currents. A similar transmission pattern was observed in the Atlantic Forest of Espírito Santo state (neighboring state in the north of Rio de Janeiro state) (Dario et al., 2017).

We rule out the importance of dogs in the transmission cycle of *T. cruzi* in the area because they presented only cryptic infection. Moreover, they were bred free-range and rural dogs that use a wide range of living areas, i.e., they may also have acquired the infection in a more distant area. In addition, no other mammalian species were infected, and the presence of triatomines was not reported by the locals or by the sanitary authorities. This scenario has not changed after three years.

In conclusion, we demonstrated that even highly degraded areas may maintain a high diversity of trypanosomatid species being transmitted. The *T. cruzi* clade proved to be increasingly diverse, and certainly, in the future, more species and/or genotypes will be revealed within this group. Finally, the acute CD case was an unfortunate coincidence because no other case has been reported.

## Data Availability Statement

The datasets presented in this study can be found in online repositories. The names of the repository/repositories and accession number(s) can be found in the article/supplementary material.

## Ethics Statement

The animal study was reviewed and approved by the Ethical Committee for Animal Use of the Oswaldo Cruz Foundation (license LW-81-12). The capture of small sylvatic mammals was authorized by the Sistema de Autorizaçao e Informaçao em Biodiversidade—SISBIO of the Instituto Brasileiro do Meio Ambiente e dos Recursos Naturais Renováveis (IBAMA)(permanent license number 3365-1) and by the Instituto Estadual do Meio Ambiente (INEA/RJ) (license number 028/2015).

## Author Contributions

AJ and AR contributed to the conception and design of the study. MD, CL, SX, PD’A, AR, and AJ undertook the research and analyses. MD and AJ wrote the draft of the manuscript. All authors contributed to the article and approved the submitted version.

## Funding

This study was funded by the Oswaldo Cruz Foundation, Conselho Nacional de Desenvolvimento Científico e Tecnológico (CNPq) and the Fundação de Amparo à Pesquisa do Estado do Rio de Janeiro (Faperj). MD receives a postdoctoral fellow by Faperj (E-26/202.414/2019). AR is financially supported by CNPq/Universal (425293/2018-1) and Jovem Cientistas do Nosso Estado/Faperj (E-26/202.794/2019). AJ is financially supported by CNPq (Bolsista de Produtividade, nível 1A). SCdCX received a financial support from CNPq/Universal (422489/2018-2).

## Conflict of Interest

The authors declare that the research was conducted in the absence of any commercial or financial relationships that could be construed as a potential conflict of interest.

## Publisher’s Note

All claims expressed in this article are solely those of the authors and do not necessarily represent those of their affiliated organizations, or those of the publisher, the editors and the reviewers. Any product that may be evaluated in this article, or claim that may be made by its manufacturer, is not guaranteed or endorsed by the publisher.
